# Structural studies on encapsulation of tetrahedral and octahedral anions by a protonated octaaminocryptand cage

**DOI:** 10.3762/bjoc.5.41

**Published:** 2009-08-31

**Authors:** I Ravikumar, P S Lakshminarayanan, E Suresh, Pradyut Ghosh

**Affiliations:** 1 Department of Inorganic Chemistry, Indian Association for the Cultivation of Science, 2A & 2B Raja S. C. Mullick Road, Kolkata 700 032, India, Fax: (+91) 33-2473-2805; 2 Analytical Science Discipline, Central Salt & Marine Chemicals Research Institute, G. B. Marg, Bhavnagar 364 002, India

**Keywords:** anion receptor, cryptand, molecular recognition, proton cage

## Abstract

Structural aspects of the binding of inorganic anions such as perchlorate, hydrogen sulfate, and hexafluorosilicate with the proton cage of octaaminocryptand **L^1^**, N(CH_2_CH_2_NHCH_2_-*p*-xylyl-CH_2_NHCH_2_CH_2_)_3_N), are examined thoroughly. Crystallographic results for a hexaprotonated perchlorate complex of **L^1^**, [(H_6_L^1^)^6+^(ClO_4_^−^)]5(ClO_4_^−^)·11H_2_O·CH_3_CN (**1**), an octaprotonated hydrogen sulfate complex of **L^1^**, [(H_8_**L^1^**)^8+^(HSO_4_^−^)]7(HSO_4_^−^)·3H_2_O·CH_3_OH (**2**) and an octaprotonated fluorosilicate complex of **L^1^**, [(H_8_**L^1^**)^8+^(HSiF_6_^−^)]3(SiF_6_^2−^)·(HSiF_6_^−^)·15H_2_O (**3**), show encapsulation of one perchlorate, hydrogen sulfate and hexafluorosilicate, respectively inside the cage of **L^1^** in their protonated states. Further, detailed structural analysis on complex **1** reveals that the hexaprotonated **L^1^** encapsulates a perchlorate *via* two N–H···O and five O–H···O hydrogen bonds from protonated secondary nitrogen atoms of **L^1^** and lattice water molecules, respectively. Encapsulated hydrogen sulfate in complex **2** is “glued” inside the octaprotonated cage of **L^1^** *via* four N–H···O and six C–H···O hydrogen bonds whereas encapsulated HSiF_6_^−^ in complex **3** has short contacts *via* six N–H···F and three C–H···F hydrogen bonds with [H_8_**L^1^**]^8+^. In the cases of complexes **2** and **3**, the cryptand **L^1^** in octaprotonated state shows monotopic encapsulation of the guest and the final conformation of these receptors is spherical in nature compared to the elongated shape of hexaprotonated state of **L^1^** in complex **1**.

## Introduction

**Figure 1:**
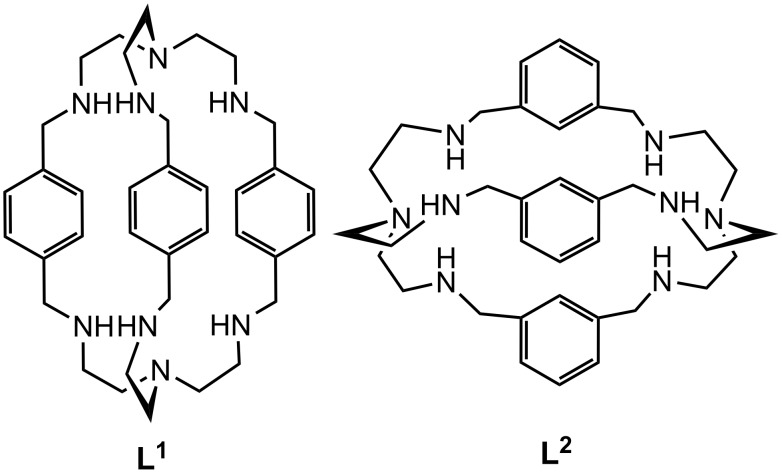
Octaaminocryptand with *p*-xylyl spacers **L^1^**, with *m*-xylyl spacers **L^2^**.

In recent years considerable efforts have been made in elucidating the coordination chemistry of anions because of their vital roles in biological systems [1], medicine [2], catalysis [3], and environmental issues [4]. Perchlorate is harmful to human health and has applications in defense [5], commercial and domestic purposes [6], whereas sulfate recognition is of current interest due to its biological [7] and environmental importance [8]. It has been observed that protonated amines and quaternary ammonium functions incorporated in a suitable ligand topology make them attractive receptors for anions [1–4]. Azamacropolycycles **L^1^** and **L^2^** (Figure 1) have shown encapsulation of different anions in their protonated states [9–22]. For example, azamacropolycycle **L^1^** (Figure 1) forms a fluoride-based cascade complex [9], whilst for chloride/bromide encapsulation inside the cavity of hexaprotonated **L^1^**, [H_6_**L^1^**]^6+^ leads to both monohydrated complexes [10] and monotopic chloride/bromide complexes [11]. Protonated ligand [H_7_**L^1^**]^7+^ leads to monotopic encapsulation of chloride *via* hydrogen bonding with external undecameric water clusters [12] whilst iodide encapsulation has been observed in the case of [H_8_**L^1^**]^8+^ [13]. Whereas there are a large number of reports on halide encapsulation in different protonated states for **L^1^**, encapsulation of polyatomic anions such as tetrahedral (ClO_4_^−^, HSO_4_^−^, H_2_PO_4_^−^), and octahedral (SiF_6_^2−^, PF_6_^−^) anions etc. have not been reported with this system, although planar (NO_3_^−^) encapsulation and binding of H_2_PO_4_^−^ by [H_6_**L^1^**]^6+^ have been observed [14–15]. By contrast, **L^2^**, as host has been extensively used for oxyanion binding [16–22]. In 1995 the first structurally characterized encapsulated ClO_4_^−^ and SiF_6_^2−^ by hexaprotonated furan and pyridine analogues of **L^1^**, respectively were reported by Nelson et al. [23]. Very recently, Bowman-James et al. have shown encapsulation of sulfate inside the cavity of [H_6_**L^2^**]^6+^ [24]. Other organic receptors for perchlorate [25–26] and sulfate [27–32] have been described in the literature. Nelson et al. have reviewed the recognition of oxanions by different azacryptand hosts [33]. Steed et al. have reported a macrobicyclic azaphane receptor for halide binding through C–H···X^−^ and N–H···X^−^ interactions [34]. In this article we report solid state structural evidence of encapsulation and binding of tetrahedral oxyanions ClO_4_^−^ and HSO_4_^−^ as well as encapsulation of octahedral anion HSiF_6_^−^ with **L^1^** in different protonated states.

## Results and Discussion

**Syntheses.** The cryptand **L^1^** was prepared on multi-gram scale and in very high yield following the modified literature procedure [13]. The key step in the scaled-up synthesis of this octaazacryptand is the condensation of tris(2-aminoethyl)amine (tren) with terephthaldehyde at 5–10 °C by the slow addition of a dry methanolic tren solution to the aldehyde also dissolved in dry methanol. Reduction of the resulting Schiff base was achieved using NaBH_4_. Both higher temperatures (40–50 °C) and fast addition rates lead to mostly polymeric products in the scaled-up synthesis. In the case of **1**, a white precipitate is obtained after addition of perchloric acid to the methanolic solution of **L^1^**, which after crystallization from acetonitrile/water (1:1 v/v), gave perchlorate encapsulated in a [H_6_**L^1^**]^6+^ cage. Complex **2** is obtained as white solid upon reacting sulfuric acid with **L^1^** in acetonitrile medium followed by crystallization from water/MeOH (1:1 v/v). Complex **3** is obtained as a white precipitate upon treating the receptor with hydrofluoric acid in methanol followed by crystallization from water. The syntheses of the complexes are all straight forward and, with the exception of complex **3**, are obtained in high yield.

**Figure 2:**
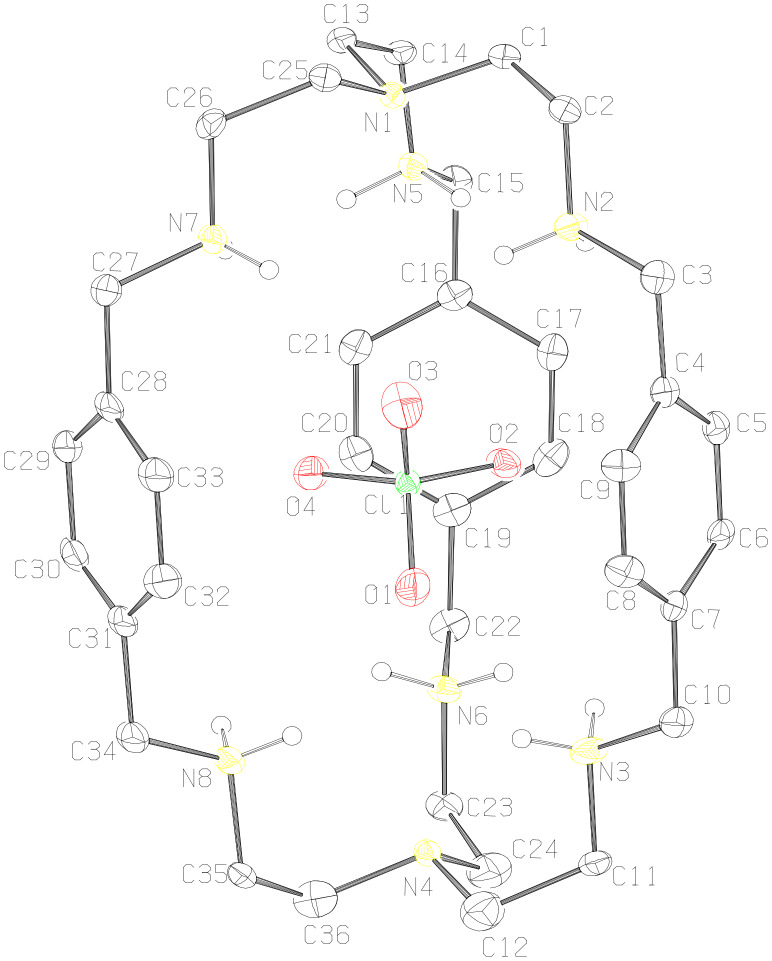
ORTEP diagram of the [H_6_**L^1^**]^6+^ with encapsulated ClO_4_^−^ (40% probability factor for the thermal ellipsoids and hydrogen atoms attached to the protonated nitrogen atoms only are shown for clarity).

**Description of the Crystal Structure,** [(H_6_**L^1^**)^6+^(ClO_4_^−^)][5(ClO_4_^−^)·11H_2_O·CH_3_CN (**1**). Hexaprotonated cryptand cage [H_6_**L^1^**]^6+^ shows encapsulation of one perchlorate ion in the cavity. This represents monotopic recognition of perchlorate whereas five perchlorate counter anions, along with eleven molecules of water and one acetonitrile molecule as solvent of crystallization are present in the lattice. The ORTEP diagram of the hexaprotonated cryptand moiety with the encapsulated perchlorate is shown in Figure 2. Here the [H_6_**L^1^**]^6+^ moiety has an *endo-endo* conformation with a distance of 9.850 Å between the two bridgehead nitrogen atoms (N1 and N4). The window between three phenyl rings ranges from 6.815 Å to 7.126 Å (measured by the centroid of phenyl distance) with an average window of 6.993 Å indicates the elliptical nature of the perchlorate encapsulated [H_6_**L^1^**]^6+^ moiety (Figure 2). All the secondary amino nitrogen atoms N2, N3, N5, N6, N7 and N8, from all three strands of the cryptand moiety are protonated, which is evident by the comparatively longer C–N bond distances of these nitrogen atoms with the neighboring carbons (Table 1).

**Table 1:** Selected non-bonded distances (Å) of complex **1**.

N···N Distance [Å]	

N2···C2	1.494(8)
N2···C3	1.508(8)
N3···C10	1.484(9)
N3···C11	1.485(9)
N5···C14	1.502(8)
N5···C15	1.502(8)
N6···C22	1.493(8)
N6···C23	1.505(8)
N7···C26	1.489(8)
N7···C27	1.497(3)
N8···C34	1.497(8)
N8···C35	1.491(8)

**Figure 3:**
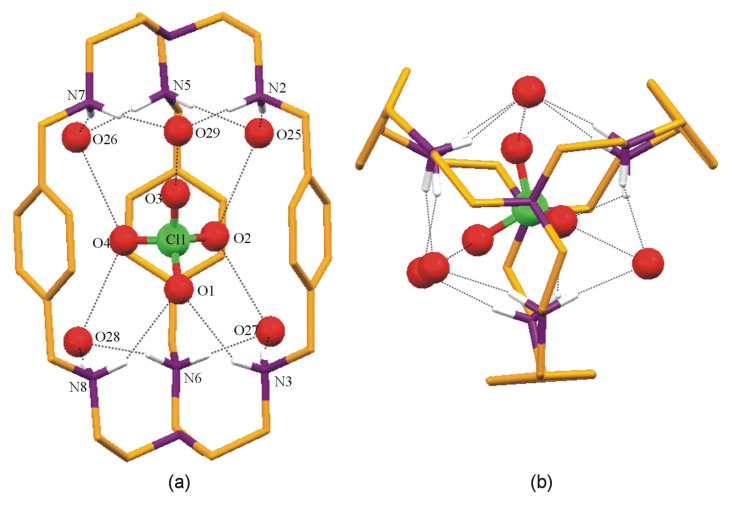
(a) Mercury diagram depicting the interactions of the encapsulated ClO_4_^−^ within the [H_6_**L^1^**]^6+^ and the surrounding water molecules. (b) Mercury diagram depicting the interactions of the encapsulated ClO_4_^−^ within the hexaprotonated tere-cryptand moiety and the surrounding water molecules viewed down the bridgehead nitrogen atoms. Hydrogen atoms other than acidic, external perchlorates and lattice water molecules are omitted for clarity.

The encapsulated perchlorate is involved in two N–H···O and five O–H···O hydrogen bonding interactions with the protonated amino hydrogen atoms and lattice water molecules, respectively, as depicted in Figure 3. Thus, the perchlorate oxygen O1 is involved in two weak intermolecular hydrogen bonds N–H···O with amino hydrogen atoms H3D and H8D of the protonated nitrogens (N3 and N8) of the cryptand with N···O distances of N3···O1 = 3.018(9) Å and N8···O1 = 3.146(8) Å, and N–H···O angles <N3–H3D···O1 = 118° and <N8–H8D···O1 = 137°, respectively. The lattice water molecules also play a vital role in anchoring the ClO_4_^−^ ion inside the flexible hexaprotonated cryptand moiety. Five lattice water molecules O25, O26, O27, O28 and O29, which act as donors and are involved in strong O–H···O hydrogen bonds with the encapsulated perchlorate oxygen atoms fasten the anion inside the cryptand moiety. All of these five water molecules act as acceptors and are oriented outside the cryptand leading to good hydrogen bonding *via* N–H···O with the protonated secondary amino hydrogen atoms (Table 2).

**Table 2:** Selected hydrogen-bond lengths (Å) and bond angles (°) of complex **1**.

D–H···A	D–H [Å]	H···A [Å]	D···A [Å]	D–H···A [°]

N2−H2C···O25	0.92	1.90	2.818(7)	172
N5−H5C···O25	0.92	1.98	2.855(7)	159
N5–H5D···O26	0.92	1.97	2.865(7)	163
N7–H7D···O26	0.92	2.03	2.929(7)	167
N3–H3C···O27	0.92	1.96	2.869(9)	168
N6–H6D···O27	0.92	1.96	2.845(8)	179
N6–H6C···O28	0.92	1.96	2.848(7)	162
N8–H8D···O28	0.92	1.98	2.897(9)	172
N2–H2D···O29	0.92	2.04	2.909(8)	158
N7–H7C···O29	0.92	1.93	2.840(8)	170

The weaker intermolecular N–H···O hydrogen bonds between the encapsulated perchlorate oxygen O1 and the [H_6_**L^1^**]^6+^ moiety could be attributed to the involvement of H3D and H8D (at the protonated secondary amine sites of the cryptand) *via* strong intermolecular N–H···O hydrogen bonding with the oxygen atom (O5) of lattice perchlorate (Table 3).

**Table 3:** Selected hydrogen-bond lengths (Å) and bond angles (°) of complex **1**.

D–H···A	D–H [Å]	H···A [Å]	D···A [Å]	D–H···A [°]

N3−H3D···O5^a^	0.92	2.04	2.932(9)	163
N8−H8D···O5^a^	0.92	2.26	3.063(8)	146

^a^ −x, −1/2+y, 1/2−z.

Even though the data were collected at 100 K, hydrogen atoms of the water molecules could not be located from the difference Fourier map, the interaction of these five water molecules are positioned near to the protonated amino nitrogen atoms *via* N–H···O hydrogen bonds. All five water molecules are further involved in strong O···O contact with the perchlorate oxygen atoms O2, O3 and O4 whereas perchlorate oxygen atom O1 binds with protonated secondary amino nitrogen atoms through two weak N–H···O hydrogen bonds which fix the ClO_4_^−^ inside the protonated cryptand moiety. As mentioned above, O1 of the ClO_4_^−^ is involved only in two weak N–H···O hydrogen bonds with the amino nitrogen atoms, whereas O2 makes short contact with O25 and O27 at distances of 2.815, and 2.804 Å, O3 with O29 at a distance of 2.806 Å and O4 with O26, and O28 at distances of 2.861 and 2.901 Å, respectively. In fact, water molecules act as donors to fix the anion inside the cavity. The concomitant effect of the weak N–H···O hydrogen bonds by the hexaprotonated cryptand moiety and the orientation of the water molecules surrounding the protonated secondary amine followed by their short contacts with the other ClO_4_^−^ ions pave way for the encapsulation of ClO_4_^−^ in the cryptand cavity. Further, in [H_6_**L^1^**]^6+^ moiety of **1**, the distances between any two of the secondary nitrogen atoms differ marginally in the two sets of tren cavities (N1N2N5N7) and (N3N4N6N8) (Table 4). This indicates that 3-fold symmetry about the axis passing through N1 and N4 is present in the solid state. The Cl1 of encapsulated perchlorate is sitting within the bridgehead plane (N1 and N4) and the C11 is placed closer to N4 (C11···N4 = 4.813Å) compared to the other bridgehead nitrogen N1 (C11···N1 = 5.037 Å). The distance between the bridgehead nitrogen atoms in **1** is 1.245 Å shorter than the distance observed in the free cryptand **L^1^** (11.095 Å) but the distance in complex **1** is 3.364 Å longer than that of the monotopic bromide complex of **L^1^** and only 0.527 Å smaller than the ditopic bromide and water in [H_6_**L^1^**]^6+^ complex reported recently [10–11]. This observation suggests that depending upon guest(s), the cavity dimension of hexaprotonated **L^1^** could change abruptly indicating the highly flexible nature of **L^1^** in its hexaprotonated state.

**Table 4:** Selected non-bonded distance (Å) of complex **1**.

N···N Distance [Å]	

N2···N5	4.530
N2···N7	4.385
N5···N7	4.548
N3···N6	4.529
N3···N8	4.427
N6···N8	4.474

**Figure 4:**
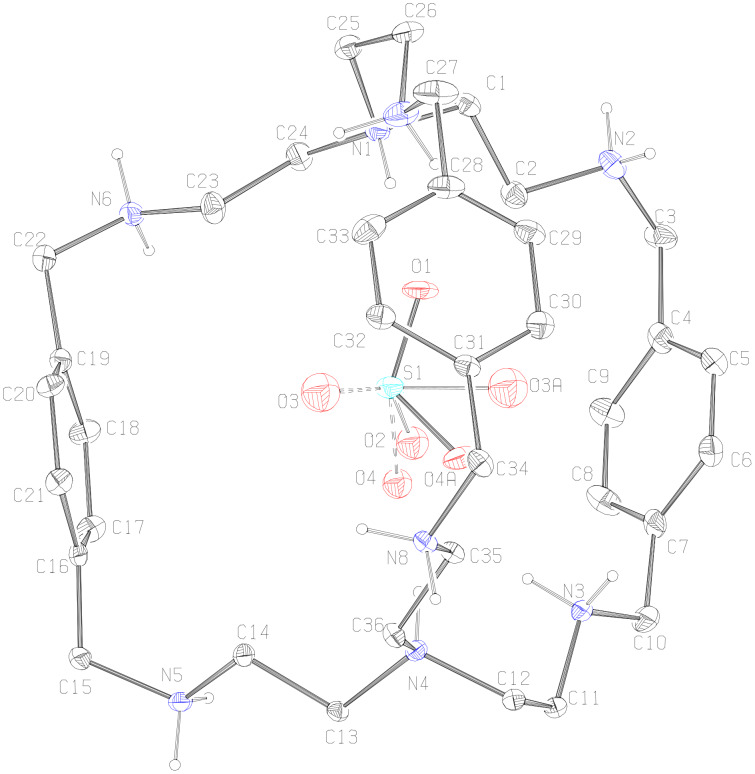
ORTEP diagram with atom numbering scheme depicting the octaprotonated **L^1^** with disordered HSO_4_^−^ monoanion inside the cavity (25% probability factor for the thermal ellipsoids and only hydrogen atoms attached to the amino nitrogens are shown in the figure for clarity).

**Description of the Crystal Structure,** [(H_8_**L^1^**)^8+^(HSO_4_^−^)]7(HSO_4_^−^)·3H_2_O·CH_3_OH (**2**). In this complex octaprotonated cryptand moiety acts as a cation and the eight [HSO_4_]^−^ anions present compensate the charge. Three molecules of water and one molecule of methanol are present in the lattice. The ORTEP diagram of the [H_8_**L^1^**]^8+^ moiety with the encapsulated HSO_4_^−^ is depicted in Figure 4. The sulfur atom S1 of the encapsulated HSO_4_^−^ deviates by 0.202 Å with respect to the plane containing the protonated apical nitrogen atoms N1 and N4. In solid state [H_8_**L^1^**]^8+^ has also an *endo-endo* conformation with a distance of 7.758 Å between two bridgehead nitrogen atoms (N1 and N4) and the window between three phenyl rings ranges from 8.099 Å to 8.403 Å (measured by the centroid of the phenyl distance) with an average window of 8.255 Å indicating the near spherical nature of the hydrogen sulfate encapsulated [H_8_**L^1^**]^8+^ moiety. The bridgehead nitrogen atoms distance in complex **2** is 2.092 Å smaller than that in complex **1** although in both cases recognition of oxyanion is monotopic in nature. This difference in complexes **1** and **2** could be due to the different degree of protonation. In fact our recent study on iodide encapsulation by [H_8_**L^1^**]^8+^ moiety shows that the bridgehead nitrogen distance in octaprotonated **L^1^** is 6.925 Å closer to the value observed in case of **2** [13]. The relatively higher value in case of complex **2** compared with the iodide encapsulated octaprotonated **L^1^** can be attributed to the polyatomic nature of HSO_4_^−^ and flexible nature of the [H_8_**L^1^**]^8+^ moiety. The sulfur atom S1 of the encapsulated HSO_4_^−^ is located at distance of 3.92 Å and 3.85 Å from N1 and N4, respectively where N4 is slightly closer to S1. In [H_8_**L^1^**]^8+^ the distances between any two of the secondary nitrogen atoms differ in the two sets of N4 cavities (N1N2N6N7) and (N3N4N5N8) in the cryptand (Table 5). This indicates that the 3-fold symmetry about the axis passing through N1 and N4 is lost in the solid state.

**Table 5:** Selected non-bonded distance (Å) of complex **2**.

N···N Distance [Å]	

N2···N6	6.548
N2···N7	5.791
N6···N7	5.809
N3···N5	5.732
N3···N8	6.078
N5···N8	6.588

**Figure 5:**
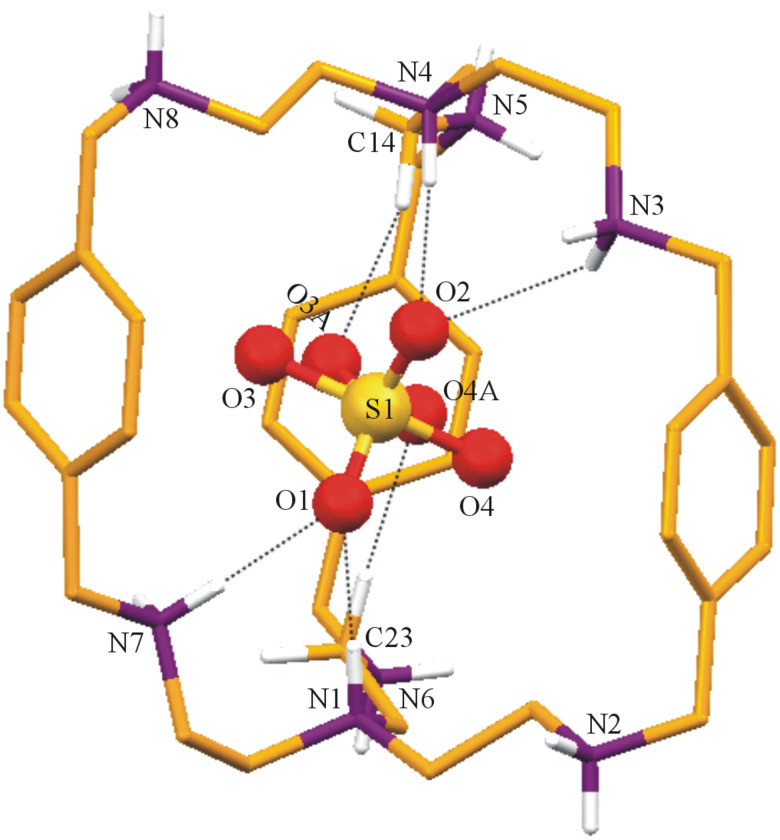
Mercury diagram depicting the encapsulation of disordered hydrogen sulfate in the cavity of [H_8_**L^1^**]^8+^ through various hydrogen bonding interactions.

Figure 5 represents the interaction of the [H_8_**L^1^**]^8+^ receptor with the encapsulated disordered hydrogen sulfate. The anion is “glued” inside the receptor by two C–H···O hydrogen bonds between the methylene hydrogen atoms (H14A, H23B) with the disordered oxygen atoms O3A and O4A, respectively, and four N–H···O contacts involving the both the protonated apical hydrogen atoms (H1D, H4D) and the hydrogen atoms (H3C and H7D) of protonated secondary amino nitrogen with O1 and O2 as acceptors each make two hydrogen bonds. Details of these intermolecular contacts are given in Table 6.

**Table 6:** Selected hydrogen-bond lengths (Å) and bond angles (°) of complex **2**.

D–H···A	D–H [Å]	H···A [Å]	D···A [Å]	D–H···A [°]

N1−H1D···O1	0.91	1.90	2.809(7)	178
N4−H4D···O2	0.91	2.02	2.896(7)	162
N3–H3C···O2	0.90	2.35	2.931(8)	123
N7–H7D···O1	0.90	2.06	2.826(10)	142
C14–H14A···O3A	0.97	2.43	3.360(2)	160
C23–H23B···O4A	0.97	2.37	3.320(17)	167

**Figure 6:**
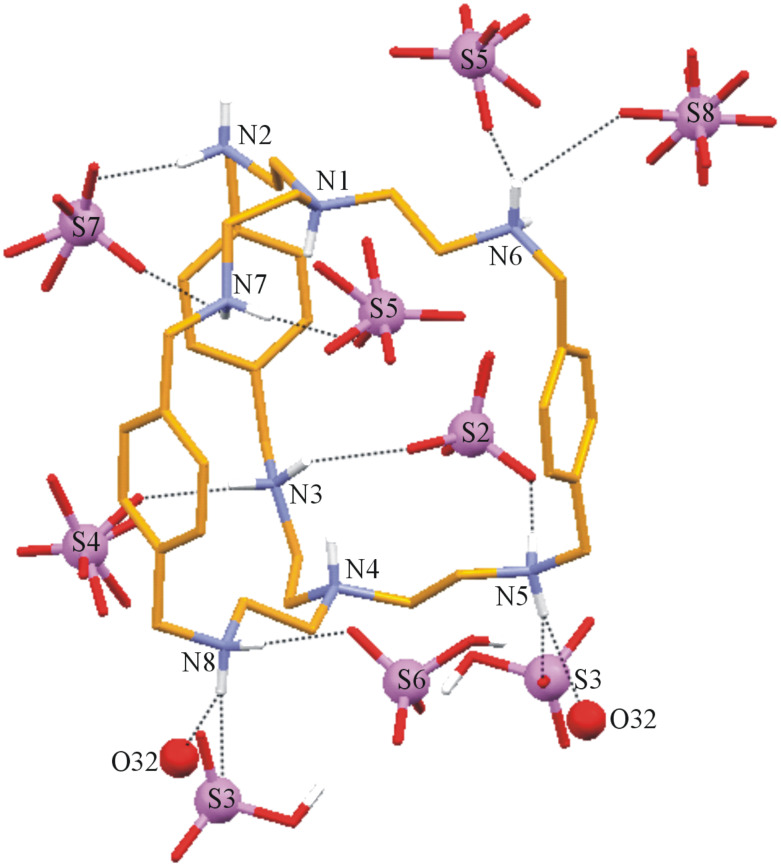
Interactions of the protonated amino nitrogen centers of the [H_8_**L^1^**]^8+^ moiety with the surrounding hydrogen sulfate and water molecules.

Figure 6 represents the additional interactions of the ammonium hydrogen atoms with the surrounding anions and water molecules. It is observed that with the exception of the apical amino hydrogen atoms all others are involved in N–H···O interactions with the lattice HSO_4_^−^ or O32 of the water molecules. Thus, hydrogen atoms attached to N5 and N8 are involved in three contacts; one with water oxygen O32 and the other two with the oxygen atoms of HSO_4_^−^ (O8, O10 for N5 and O10, O21 for N8). The rest of the ammonium hydrogen atoms are also involved in effective N–H···O contacts with the hydrogen sulfate as depicted in Figure 6.

**Figure 7:**
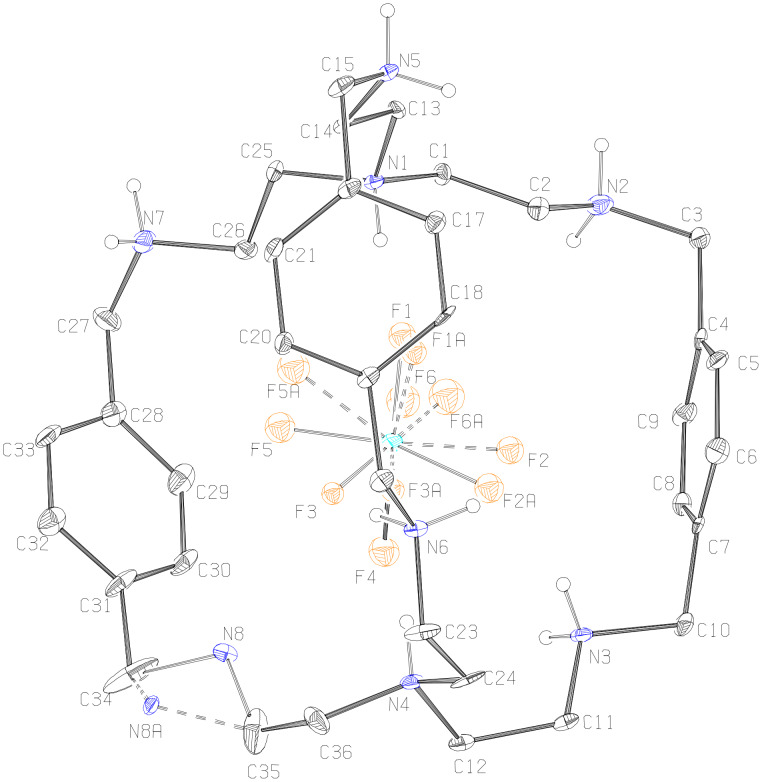
ORTEP diagram depicting the octaprotonated [H_8_**L^1^**]^8+^ moiety with the encapsulated disordered HSiF_6_^−^ monoanion with atom numbering scheme (25% probability factor for the thermal ellipsoids and only hydrogen atoms attached to the amino nitrogens are shown for clarity).

**Figure 8:**
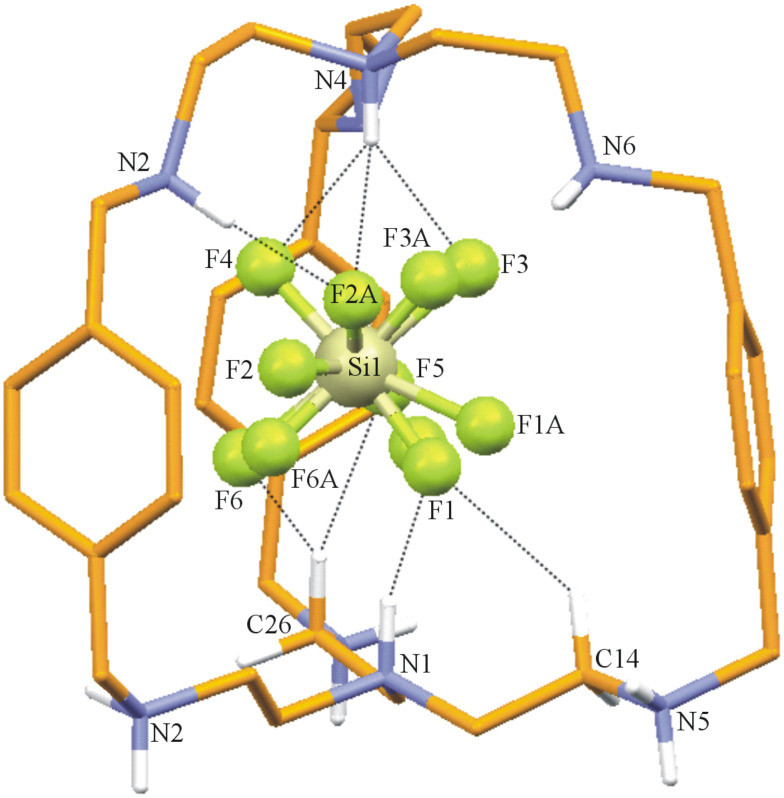
Mercury diagram depicting the encapsulation of the disordered HSiF_6_^−^ inside the [H_8_**L^1^**]^8+^ moiety along with various hydrogen bonding interactions. Only hydrogen atoms having interactions with encapsulated anion are shown for clarity.

**Description of the Crystal Structure,** [(H_8_**L^1^**)^8+^(HSiF_6_^−^)]3(SiF_6_^2−^)·(HSiF_6_^−^)·15H_2_O (**3**). Silicon hexafluoride salt of **L^1^** is obtained on reaction between **L^1^** and HF, apparently as a result of glass corrosion. The salt [H_8_**L^1^**]^8+^ has three molecules of SiF_6_^2−^, and two molecules of HSiF_6_^−^ anions to compensate the charge and fifteen water molecules as solvent of crystallization. The ORTEP diagram of the octaprotonated cryptand with the encapsulated disordered HSiF_6_^−^ monoanion is depicted in Figure 7 and the various interactions of the disordered HSiF_6_^−^ monoanion with the host molecule is depicted in Figure 8. Thus, hydrogen atoms H1 and H4 attached to the apical nitrogen N1 and N4 form N–H···F hydrogen bonds (one and three) with F1 and F2A, F3, F4, respectively. Both F2A and F3 are involved in an additional N–H···F hydrogen bonding interaction with the protonated secondary amino hydrogen atoms H3D and H6D attached to N3 and N6, respectively. F1 of the disordered encapsulated HSiF_6_^−^ is involved in intermolecular C–H···F contacts with the methylenic hydrogen atom H14B, while H26B of the methylene hydrogen attached to C26 forms bifurcated weak C–H···F hydrogen bonds [35–38] with F5 and F6 in fixing the monoanion inside the cryptand moiety (Figure 8). Details of these hydrogen bonding interactions are given in Table 7. The C–N distances involving the amino nitrogen range from 1.49 to 1.53 Å clearly indicate the octa protonation of the cryptand moiety including both the apical nitrogen atoms and are well within the range of earlier reported values [13]. Protonation of the SiF_6_^2−^ is clearly reflected in the case of Si1 and Si3 by the longer Si–F distances: Si(1)–F(4) = 1.725(5) Å, and Si(3)–F(14) = 1.742(6) Å, indicating that the encapsulated anion is HSiF_6_^−^. The Si1 of encapsulated HSiF_6_^−^ monoanion is slightly above by 0.89 Å from the plane involving the apical protonated nitrogen atoms with N1–Si1 distance of 3.854 Å and a N4–Si1 distance of 3.739 Å, respectively. In the solid state [H_8_**L^1^**]^8+^ has also an *endo-endo* conformation with a distance of 7.571 Å between the two bridgehead nitrogen atoms (N1 and N4) and the window between three phenyl rings ranges from 8.246 Å to 8.368 Å (measured by the centroid of phenyl distance) with an average window of 8.346 Å which is very close to the distances observed in complex **2** where **L^1^** is also in octaprotonated state.

**Table 7:** Selected hydrogen-bond lengths (Å) and bond angles (°) of complex **3**.

D–H···A	D–H [Å]	H···A [Å]	D···A [Å]	D–H···A [°]

N1−H···F1	0.91	1.88	2.756(9)	161
N3−H3D···F2A	0.90	1.92	2.789(11)	163
N4–H4···F2A	0.91	2.27	3.053(11)	143
N4–H4···F3	0.91	2.12	2.904(10)	143
N4–H4···F4	0.91	2.25	2.988(8)	137
N6–H6D···F3	0.90	1.86	2.726(8)	160
C14–H14B···F1	0.97	2.41	3.190(10)	137
C26–H26B···F5	0.97	2.39	3.318(14)	161
C26–H26B···F6	0.97	2.45	3.180(14)	132

**Figure 9:**
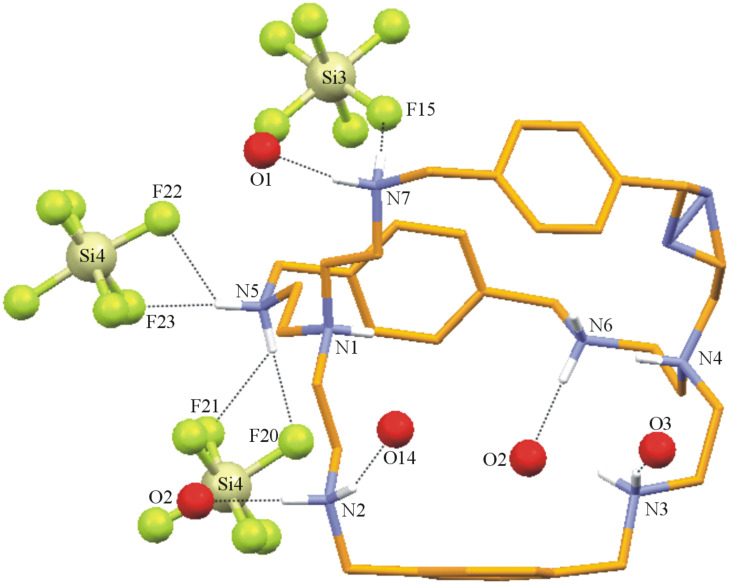
Mercury diagram depicting the interaction of the [H_8_**L^1^**]^8+^ with the surrounding molecules *via* N–H···F and N–H···O hydrogen bonds.

Figure 9 represents the interaction of the protonated amino nitrogen atoms with the molecules surrounding the moiety. As depicted in the figure the hydrogen atoms of protonated secondary nitrogen centers are involved in strong N–H···F and N–H···O hydrogen bonds with the external anions and lattice water molecules.

## Conclusions

The structural results for the interaction of polyatomic anions with the ligand **L^1^** in its hexa and octa protonated states show some interesting results. The structures clearly illustrate the effect of hexaprotonation and octaprotonation on the encapsulation of different anions. Upon a higher degree of protonation (hexa and octa) distribution of positive charge over the receptor increases which makes the cavity more electrophilic. Different degrees of protonation also change the overall conformation (ellipsoid and near spherical), which allows encapsulation of anions like perchlorate, hydrogen sulfate and hexafluorosilicate inside the receptor. Furthermore, these results indeed show that **L^1^** is also a potential receptor for bigger polyatomic anions like perchlorate and hydrogen sulfate.

## Supporting Information

Experimental procedures, characterization data and copies of spectra (^1^H NMR and HRMS) of complexes **1**, **2**, and **3** as well as crystallographic data and tables of hydrogen bonding parameters of complexes **1**, **2**, and **3** are provided.

File 1 Experimental and analytical data.
