# Supplementary material for: Structural studies on encapsulation of tetrahedral and octahedral anions by a protonated octaaminocryptand cage
Source: Beilstein J Org Chem. 2009 Aug 31;5:41. doi: 10.3762/bjoc.5.41 (PMC2779740; doi:10.3762/bjoc.5.41)
Supplement: File 1 — Experimental and analytical data. [file Beilstein_J_Org_Chem-05-41-s001.doc]

**Supporting Information**

# Structural studies on encapsulation of tetrahedral and octahedral anions by a protonated octaaminocryptand cage

I. Ravikumar^1^, P. S. Lakshminarayanan^1^, E. Suresh^*, 2^ and Pradyut Ghosh^*, 1^

Address:^1^Department of Inorganic Chemistry, Indian Association for the Cultivation of Science, 2A & 2B Raja S. C. Mullick Road, Kolkata 700 032, India, Fax: (+91) 33-2473-2805 and ^2^Analytical Science Discipline, Central Salt & Marine Chemicals Research Institute, G. B. Marg, Bhavnagar 364 002, India.

Email: Pradyut Ghosh - icpg@iacs.res.in, E. Suresh - esuresh@csmcri.org

*Corresponding author

**Experimental Section**

**Materials.** Terephthaladehyde, and tris (2-aminoethyl) amine (tren) were purchased from Sigma-Aldrich and used as received. Solvents, sodium borohydride, perchloric acid (70%), sulfuric acid (98%), and hydrofluoric (48%) acid were purchased from SD Fine Chemicals, Mumbai, India. All the solvents were purified prior to use.

**Physical Measurements.** ^1^H NMR spectra for complexes were recorded on Bruker 300 MHz FT-NMR spectrometers (model: DPX-300) instrument in DMSO-*d_6_* at 25 ºC. HRMS measurements were carried out on QTof-Micro YA 263 instruments in positive ESI mode.

**Synthesis of complex** [(H_6_**L^1^**)^6+^(ClO_4_^−^)][5(ClO_4_^−^)·11H_2_O·CH_3_CN (**1**). Complex **1** was obtained by dissolving **L^1^** (60 mg, 1 mmol) in MeOH (10 ml) and adding 70% HClO_4_. The precipitate formed was re-dissolved in 10 ml of H_2_O:CH_3_CN mixture (1:1 v/v) and filtered. Colorless crystals suitable for X-ray analysis were grown after a week by slow evaporation. Yield: 75%. ^1^H NMR (300 MHz, DMSO-*d_6_*): δ 2.63 (br, 12H, NC*H_2_*), 2.79 (br, 12H, NCH_2_C*H*_2_), 4.29 (s, 12H, ArC*H*_2_), 7.52 (s, 12H, Ar*H*), 8.64 (br, 12H, -N*H*). HRMS (ESI): m/z 599.8494 [H**L^1^**]^+^, 699.8786 [(H_2_**L^1^**)^2+^+ClO_4_^−^], 799.8772 [(H_3_**L^1^**)^3+^ +2·ClO_4_^−^], 899.8885 [(H_4_**L^1^**)^4+^ + 3·ClO_4_^−^].

**Synthesis of complex** [(H_8_**L^1^**)^8+^(HSO_4_^−^)][7(HSO_4_^−^)·3H_2_O·CH_3_OH (**2**). Complex **2** was obtained by dissolving **L^1^** (60 mg, 1 mmol) in CH_3_CN (10 ml) and adding 98% H_2_SO_4_. The precipitate formed was re-dissolved in 10 ml of water:MeOH mixture (1:1 v/v) and filtered. The filtrate was allowed to evaporate slowly at room temperature. Colorless crystals suitable for X-ray analysis were grown after 10 days. Yield: 60%. ^1^H NMR (300 MHz, DMSO-*d_6_*): δ 2.72 (br, 12H, NC*H_2_*), 2.97 (br, 12H, NCH_2_C*H*_2_), 4.24 (s, 12H, ArC*H*_2_), 7.65 (s, 12H, Ar*H*), 8.44 (br, 12H, -N*H*). HRMS (ESI): m/z 599.3102 [H**L^1^**]^+^, 697.2662 [(H_2_**L^1^**)^2+^ +HSO_4_^−^], 795.1293 [(H_3_**L^1^**)^3+^ + 2·HSO_4_^−^].

**Synthesis of complex** [(H_8_**L^1^**)^8+^(HSiF_6_^−^)]3(SiF_6_^2−^)·(HSiF_6_^−^)·15H_2_O (**3**). Complex **3** was obtained by dissolving **L^1^** (60 mg, 1 mmol) in MeOH (10 ml) and adding 48% HF in a glass beaker. The precipitate formed was re-dissolved in 10 ml of water, warmed and filtered. The filtrate was allowed to evaporate slowly at room temperature. Colorless crystals suitable for X-ray analysis were grown after 3 days. Yield: 35%. ^1^H NMR (300 MHz, DMSO-*d_6_*): δ 2.68 (br, 12H, NC*H_2_*), 2.91 (br, 12H, NCH_2_C*H*_2_), 4.25 (s, 12H, ArC*H*_2_), 7.63 (s, 12H, Ar*H*), 8.34 (br, -N*H*). HRMS (ESI): m/z 599.2839 [H**L^1^**]^+^, 687.2738 [(H_2_**L^1^**)^2+^ + HSiF_6_^−^].

**X-ray Crystallography.** The crystallographic data and details of data collection for all the complexes **1**, **2**, and **3** are given in Table 1S. The crystals of all three complexes were very unstable on exposure to the atmosphere and hence a crystal of suitable size was selected from the mother liquor, immersed immediately in paratone oil and mounted on the tip of a glass fiber, and cemented using epoxy resin. Intensity data for all three crystals were collected using MoK_α_ (λ = 0.71073Å) radiation on a Bruker SMART APEX diffractometer equipped with CCD area detector at 100 K. The data integration and reduction were processed with SAINT [1] software. An empirical absorption correction was applied to the collected reflections with SADABS [2]. The structure was solved by direct methods using SHELXTL [3] and refined on *F^2^* by the full-matrix least-squares technique using the SHELXL-97 [4] package.

In complex **1** the non-hydrogen atoms of the ligand moiety, and the five perchlorate anions, lattice water molecules and acetonitrile molecule were refined anisotropically until convergence was reached. In case of one perchlorate anion in the lattice, two oxygen atoms are disordered at two sites and the occupancy factor for these oxygen atoms with dynamic disorder (O22 and O24) were determined using the FVAR command of the SHELXTL program. These atoms were refined only isotropically in the final cycles of refinement. Even although, the data were collected at 100 K, we could not locate hydrogen atoms attached to the lattice water molecules and hydrogen atoms attached to the ligand moiety as well as the acetonitrile molecules are sterochemically fixed.

For complex **2,** non-hydrogen atoms of the ligand moiety, two HSO_4_^−^ anions, three water molecules and the one methanol molecule located, were refined anisotropically until convergence was reached. During the refinement process, it was observed that with the exception of two HSO_4_^−^anions, in the other six HSO_4_^−^ anions more than one oxygen atom attached to the sulfur has partial occupancy at two positions and the occupancy factor of the disordered atoms were fixed by the FVAR command of the SHELXTL program, and refined only isotropically. In this case the data were also collected at 100 K however, we could not locate/ fix the hydrogen atoms attached to the anions due to severe disorder of the oxygen atoms of hydrogen sulfate group in six anions and the lattice water molecules. The protonation of these partly occupied disordered anions were uncertain and hydrogen atoms were fixed and refined only for two hydrogen sulfate anions with full occupancy factor. In order to compensate the charge of the octaprotonated cryptand, it is assumed that all the eight hydrogen sulfate groups may be monoprotonated i.e. in the HSO_4_^–^ form. Hydrogen atoms of the protonated ligand moiety and the lattice methanol molecules were stereochemically fixed. In addition to the three water molecules and one methanol located for the complex from the difference Fourier map, a number of diffuse scattered peaks with electron density ranging from 2.9 Å^−3^ to 2.5 Å^−3^ were observed, which can be attributed to disordered solvent present in this complex. Attempts to model these peaks were unsuccessful since residual electron density peaks obtained were diffuse and there were no obvious major site occupations for the solvent molecules. PLATON/SQUEEZE [5] was used to correct the data for the presence of the disordered solvent. A potential solvent volume of 168.9Å^3^ was found. Therefore, 60 electrons count per unit cell worth of scattering were located in the void which indicates that the void present in the unit cell can accommodate tentatively 60 electrons containing solvent. This electrons count corresponds to tentatively three methanol molecules and a water molecule present in the unit cell. Thus, the structure of the compound revealed one molecule of [H_8_**L^1^**]^8+^, eight HSO_4_^−^ ions, 3 lattice water molecules and one methanol in the asymmetric unit along with tentatively 3 more molecules of disordered methanol and one more water molecule in the lattice, whose contribution was removed by PLATON/SQUEEZ program [5]. With the modified data set using SQUEEZ program, the final cycles of least-squares refinements improved both the R-values and Goodness of Fit significantly.

In complex **3** non-hydrogen atoms of the ligand moiety, four anions, and the lattice water molecules were refined anisotropically until convergence was reached. In the encapsulated HSiF_6_^2–^, five out of the six fluorine atoms showed dynamic disorder at two different positions and the occupancy factor of the disordered atoms were fixed by the FVAR command of the SHELXTL program and refined only isotropically. One of the nitrogen atoms of the cryptand moiety is also disordered and FVAR command was used to fix the occupancy factor and these atoms were refined anisotropically. Although the data was collected at 100 K, we could not locate hydrogen atoms attached to the lattice water molecules, disordered nitrogen N8 of the ligand moiety and two of the SiF_6_^2−^ anions, whilst the rest of the hydrogen atoms were sterochemically fixed. In addition to the fifteen water molecule located for the compound from the difference Fourier map, a number of diffuse scattered peaks with electron density ranging from 3.0 Å^−3^ to 2.5 Å^−3^ were observed, which can be attributed to disordered solvent present in this compound. Attempts made to model this were unsuccessful since residual electron density peaks obtained were diffuse and there were no obvious major site occupations for the solvent molecules. PLATON/SQUEEZE [5] was used to correct the data for the presence of the disordered solvent. A potential solvent volume of 275.5 Å^3^ was observed with 61 eÅ^3^ residual electrons counts per unit cell worth of scattering were located in the void. This residual electron counts corresponds to approximately 3 three methanol molecules and a water molecule present in the unit cell. Thus, the structure revealed that there are one molecule of octaprotonated cryptand, 5 counter ions and 15 lattice water molecules (for which the H-atoms were not located) in the asymmetric unit along with tentatively 3 molecules of disordered methanol and one more water molecule in the lattice, whose contribution was removed by PLATON/SQUEEZ program. With the modified data set using SQUEEZ program, the final cycles of least-squares refinements improved both the R-values and Goodness of Fit significantly. The diagrams of the crystal structures were generated using programs ORTEP [6], Mercury 1.4.1 [7] or PALTON [5].

**References:**

1. Sheldrick, G. M. SAINT 5.1 ed.; Siemens Industrial Automation Inc.: Madison, WI, **1995**.

2. *SADABS, empirical absorption Correction Program*; University of Göttingen: Göttingen, Germany,**1997**.

3. Sheldrick, G. M. *SHELXTL Reference Manual:* V*ersion 5.1*; Bruker AXS: Madison, WI, **1997**.

4. Sheldrick, G.M. *SHELXL-97: Program for Crystal Structure Refinement*; University of Göttingen: Göttingen, Germany, **1997**.

5. Spek, A. L. *PLATON for MS-Windows*, *J. Appl. Cryst*. 36, 7-13.

6. Farrugia, L. J. *Ortep-3 for Windows***:** *J. Appl. Cryst.* **1997**, *30*, 565.

7. Macrae, C. F.; Edgington, P. R.; McCabe, P.; Pidcock, E.; Shields, G. P.; Taylor, R.; Towler, M.; Van de Streek, J. *Mercury: visualization and analysis of crystal structures, J. Appl. Cryst.* **2006**, *39*, 453-457.

**Table 1S**: Crystallographic data for complexes **1**, **2** and **3**.

| Compound | **Complex 1**  **CCDC 710521** | **Complex 2**  **CCDC 710520** | **Complex 3**  **CCDC 710519** |
| --- | --- | --- | --- |
| empirical formula | C_38_H_63_Cl_6_N_9_O_33_ | C_37_H_68_N_8_O_34_S_7.5_ | C36H56F27N8O15Si4.5 |
| formula weight | 1386.67 | 1409.44 | 1480.29 |
| crystal system | monoclinic | Triclinic | Triclinic |
| Space group | P21/c | P-1 | P-1 |
| a (Å) | 11.3787(10) | 12.294(5) | 12.358(7) |
| b (Å) | 19.6146(18) | 12.605(5) | 13.543(8) |
| c (Å) | 27.025(3) | 23.350(9) | 22.382(14) |
| α (º) | 90.00 | 86.820(6) | 102.967(13) |
| β (º) | 96.879(2) | 87.591(7) | 90.569(13) |
| γ (º) | 90.00 | 63.858(6) | 111.774(11) |
| *V* (Å^3^) | 5988.3(9) | 3243(2) | 3372(3) |
| Z | 4 | 2 | 2 |
| *d_calc_* (g/cm^3^) | 1.538 | 1.444 | 1.458 |
| Crystal size | 0.43 x 0.24 x 0.18 | 0.34 x 0.28 x 0.22 | 0.42 x 0.36 x 0.26 |
| Diffractometer | SMART CCD | SMART CCD | SMART CCD |
| λ (Å) | 0.71073 | 0.71073 | 0.71073 |
| *F*(000) | 2880 | 1476 | 1508 |
| μ Mo Kα (mm^-1^) | 0.387 | 0.353 | 0.230 |
| Abs corr | SADABS | SADABS | SADABS |
| T (K) | 100 | 100 | 100 |
| Min. and Max. Transmission | 0.8513 and 0.9336 | 0.8895 and 0.9264 | 0.9096 and 0.9426 |
| θ (range) | 1.29-25.00 | 1.85-26.00 | 1.70-26.00 |
| Reflns collected | 28547 | 16766 | 18099 |
| Indep reflns | 10413 | 12321 | 12822 |
| *R* (int) | 0.0332 | 0.0337 | 0.0523 |
| Data/restr/param | 10413/0/785 | 12321/0/744 | 12822/0/830 |
| *R*_1_; w*R*_2_(I ≥ σ(I)) | 0.1111; 0.3068 | 0.1258; 0.3542 | 0.1166; 0.3003 |
| GOF *(F^2^)* | 1.072 | 1.252 | 1.019 |
| Residual electron density (Å^-3^) | 2.314, -1.266 | 2.421, -0.695 | 1.311, -0.703 |

**Table 2S**: Hydrogen bonding table for complex **1**.

| **D-H···A** | **D-H [Å]** | **H···A [Å ]** | **D···A [Å ]** | **D-H···A [^o^]** |
| --- | --- | --- | --- | --- |
| N(2)-H(2C)**···**O(25)^1^ | 0.92 | 1.90 | 2.818(7) | 172 |
| N(2)-H(2D)**···**O(29)^2^ | 0.92 | 2.04 | 2.909(8) | 158 |
| N(3)-H(3C)**···**O(27)^1^ | 0.92 | 1.96 | 2.869(9) | 168 |
| N(3)-H(3D)**···**O(1)^1^ | 0.92 | 2.47 | 3.0189(9) | 118 |
| N(3)-H(3D)**···**O(5)^2^ | 0.92 | 2.04 | 2.932(9) | 163 |
| N(5)-H(5C)**···**O(25)^1^ | 0.92 | 1.98 | 2.855(7) | 159 |
| N(5)-H(5D)**···**O(26)^1^ | 0.92 | 1.97 | 2.865(7) | 163 |
| N(6)-H(6C)**···**O(28)^1^ | 0.92 | 1.96 | 2.848(7) | 162 |
| N(6)-H(6D)**···**O(27)^1^ | 0.92 | 1.92 | 2.845(8) | 179 |
| N(7)-H(7C)**···**O(29)^2^ | 0.92 | 1.93 | 2.840(8) | 170 |
| N(7)-H(7D)**···**O(26)^1^ | 0.92 | 2.03 | 2.929(7) | 167 |
| N(8)-H(8C)**···**O(25)^1^ | 0.92 | 1.98 | 2.897(9) | 172 |
| N(8)-H(8D)**···**O(1)^1^ | 0.92 | 2.41 | 3.146(8) | 137 |
| N(8)-H(8D)**···**O(5)^2^ | 0.92 | 2.26 | 3.063(8) | 146 |
| C(10)-H(10A)**···**O(31)^1^ | 0.99 | 2.51 | 3.313(12) | 138 |
| C(14)-H(14B)**···**O(20)^3^ | 0.99 | 2.51 | 3.387(10) | 147 |
| C(15)-H(15A)**···**O(21)^3^ | 0.99 | 2.31 | 3.256(10) | 159 |
| C(22)-H(22A)**···**O(6)^3^ | 0.99 | 2.52 | 3.506(9) | 173 |
| C(22)-H(22B)**···**O(11)^3^ | 0.99 | 2.34 | 3.283(11) | 158 |
| C(24)-H(24A)**···**O(20)^4^ | 0.99 | 2.55 | 3.431(11) | 148 |
| C(24)-H(24B)**···**O(9)^3^ | 0.99 | 2.50 | 3.251(13) | 132 |
| C(25)-(25A)**···**O(22A)^5^ | 0.99 | 2.49 | 3.266(17) | 135 |
| C(26)-H(26A)**···**O(22)^2^ | 0.99 | 2.59 | 3.326(14) | 131 |
| C(26)-H(26B)**···**O(10)^1^ | 0.99 | 2.38 | 3.305(9) | 156 |
| C(27)-H(27A)**···**O(11)^1^ | 0.99 | 2.46 | 3.452(12) | 175 |
| C(34)-H(34B)**···**O(17)^1^ | 0.99 | 2.43 | 3.196(12) | 133 |
| C(34)-H(34B)**···**N(9)^1^ | 0.99 | 2.58 | 3.253(12) | 125 |
| C(35)-H(35A)**···**O(7)^2^ | 0.99 | 2.52 | 3.382(10) | 146 |
| C(35)-H(35B)**···**O(19)^1^ | 0.99 | 2.56 | 3.371(11) | 139 |
| C(37)-(37C)**···**O(24A)^1^ | 0.98 | 2.48 | 3.337(19) | 146 |
| 1. x, y, z; 2. 1-x, -1/2+y, -1/2-z; 3. –x, -1/2+y, -1/2-z; 4. –x, 1-y, 1-z; 5. x, 3/2-y, -1/2+z. | | | | |

**Table 3S:** Hydrogen bonding table for complex **2**.

| **D-H···A** | **D-H [Å]** | **H···A [Å ]** | **D···A [Å ]** | **D-H···A [^o^]** |
| --- | --- | --- | --- | --- |
| N(1)-H(1D)**···**O(1)^1^ | 0.91 | 1.90 | 2.809(7) | 178 |
| N(3)-H(3C)**···**O(2)^1^ | 0.90 | 2.35 | 2.931(8) | 123 |
| N(4)-H(4D)**···**O(2)^1^ | 0.91 | 2.02 | 2.896(7) | 162 |
| N(7)-H(7D)**···**O(1)^1^ | 0.90 | 2.06 | 2.826(10) | 142 |
| C(14)-H(14A)**···**O(3A)^1^ | 0.97 | 2.43 | 3.360(2) | 160 |
| C(23)-H(23B)**···**O(4A)^1^ | 0.97 | 2.37 | 3.320(17) | 167 |
| N(2)-H(2C)**···**O(26)^2^ | 0.90 | 1.89 | 2.780(13) | 169 |
| N(3)-H(3C)**···**O(5)^1^ | 0.90 | 2.06 | 2.848(8) | 145 |
| N(3)-H(3D)**···**O(15A)^3^ | 0.90 | 1.99 | 2.880(2) | 170 |
| N(5)-H(5C)**···**O(8)^1^ | 0.90 | 1.86 | 2.761(9) | 177 |
| N(5)-H(5D)**···**O(10)^4^ | 0.90 | 2.00 | 2.819(7) | 151 |
| N(5)-H(5D)**···**O(32)^4^ | 0.90 | 2.50 | 3.150(7) | 129 |
| N(6)-H(6C)**···**O(31A)^5^ | 0.90 | 2.49 | 3.07(3) | 123 |
| N(6)-H(6C)**···**O(18)^6^ | 0.90 | 1.96 | 2.756(13) | 147 |
| N(7)-H(7C)**···**O(20)^7^ | 0.90 | 1.97 | 2.871(12) | 176 |
| N(7)-H(7D)**···**O(27)^2^ | 0.90 | 2.59 | 3.211(11) | 127 |
| N(8)-H(8C)**···**O(21)^7^ | 0.90 | 2.00 | 2.873(8) | 164 |
| N(8)-H(8D)**···**O(10)^7^ | 0.90 | 2.35 | 3.007(7) | 130 |
| N(8)-H(8D)**···**O(32)^7^ | 0.90 | 2.07 | 2.862(7) | 146 |
| O(12)-H(12E)**···**O(24)^1^ | 0.82 | 1.82 | 2.587(10) | 155 |
| O(23)-H(23E)**···**O(13)^8^ | 0.82 | 2.00 | 2.648(13) | 135 |
| C(1)-H(1A)**···**O(28)^6^ | 0.97 | 2.53 | 3.150(2) | 122 |
| C(1)-H(1B)**···**O(27)^2^ | 0.97 | 2.59 | 3.260(13) | 127 |
| C(1)-H(1B)**···**O(28A)^2^ | 0.97 | 2.59 | 3.560(2) | 177 |
| C(3)-H(3A)**···**O(17)^3^ | 0.97 | 2.32 | 3.100(3) | 136 |
| C(3)-H(3B)**···**O(30)^9^ | 0.97 | 2.54 | 3.141(17) | 120 |
| C(3)-H(3B)**···**O(31)^9^ | 0.97 | 2.38 | 3.240(2) | 148 |
| C(10)-H(10B)**···**O(11)^3^ | 0.97 | 2.34 | 3.148(10) | 140 |
| C(12)-H(12A)**···**O(14)^3^ | 0.97 | 2.49 | 3.240(2) | 133 |
| C(12)-H(12B)**···**O(22)^4^ | 0.97 | 2.53 | 3.375(9) | 146 |
| C(13)-H(13A)**···**O(5)^1^ | 0.97 | 2.57 | 3.113(9) | 116 |
| C(15)-H(15B)**···**O(11)^1^ | 0.97 | 2.56 | 3.485(12) | 160 |
| C(23)-H(23A)**···**O(20)^7^ | 0.97 | 2.59 | 3.431(14) | 145 |
| C(25)-H(25A)**···**O(18)^7^ | 0.97 | 2.44 | 3.289(13) | 146 |
| C(26)-(26A)**···**O(30)^10^ | 0.97 | 2.56 | 3.465(19) | 155 |
| C(26)-(26B)**···**O(28A)^2^ | 0.97 | 2.38 | 3.345(19) | 175 |
| C(27)-(27B)**···**O(31)^11^ | 0.97 | 2.36 | 3.310(2) | 164 |
| C(34)-H(34B)**···**O(11)^7^ | 0.97 | 2.39 | 3.243(10) | 145 |
| C(36)-H(36A)**···**O(22)^7^ | 0.97 | 2.40 | 3.209(10) | 140 |
| C(36)-H(36B)**···**O(22)^4^ | 0.97 | 2.52 | 3.351(9) | 144 |
| C(37)-H(37A)**···**N(6)^1^ | 0.96 | 2.11 | 2.8056 | 128 |
| C(37)-H(37B)**···**O(29)^4^ | 0.96 | 2.27 | 3.0539 | 139 |
| 1. x, y, z; 2. 1+x, 1+y, z; 3. x, 1+y, z; 4. 1-x, 1-y, 1-z; 5. x, y, -1+z; 6. 1-x, 1-y, -z; 7. 1+x, y, z; 8. -1+x, y, z; 9. 1-x, 2-y, 1-z; 10. 1+x, y, -1+z; 11. 2-x, 1-y, 1-z. | | | | |

**Table 4S**: Hydrogen bonding table for complex **3**.

| **D-H···A** | **D-H [Å]** | **H···A [Å ]** | **D···A [Å ]** | **D-H···A [^o^]** |
| --- | --- | --- | --- | --- |
| N(1)-H(1)**···**F(1)^1^ | 0.91 | 1.88 | 2.756(9) | 161 |
| N(3)-H(3D)**···**F(2A)^1^ | 0.90 | 1.92 | 2.789(11) | 163 |
| N(4)-H(4)**···**F(2A)^1^ | 0.91 | 2.27 | 3.053(11) | 143 |
| N(4)-H(4)**···**F(3)^1^ | 0.91 | 2.12 | 2.904(10) | 143 |
| N(4)-H(4)**···**F(4)^1^ | 0.91 | 2.25 | 2.988(8) | 137 |
| N(6)-H(6D)**···**F(3)^1^ | 0.90 | 1.86 | 2.726(8) | 160 |
| C(14)-H(14B)**···**F(1)^1^ | 0.97 | 2.41 | 3.190(10) | 137 |
| C(26)-H(26B)**···**F(5)^1^ | 0.97 | 2.39 | 3.318(14) | 161 |
| C(26)-H(26B)**···**F(6)^1^ | 0.97 | 2.45 | 3.180(14) | 132 |
| N(2)-H(2C)**···**O(12)^2^ | 0.90 | 1.97 | 2.740(12) | 143 |
| N(2)-H(2D)**···**O(14)^2^ | 0.90 | 2.04 | 2.899(11) | 158 |
| N(2)-H(2C)**···**O(3)^1^ | 0.90 | 1.96 | 2.839(8) | 165 |
| N(5)-H(5C)**···**F(20)^1^ | 0.90 | 1.96 | 2.826(10) | 162 |
| N(5)-H(5C)**···**F(21)^1^ | 0.90 | 2.48 | 3.134(8) | 129 |
| N(5)-H(5D)**···**F(22)^3^ | 0.90 | 2.47 | 3.089(8) | 126 |
| N(5)-H(5D)**···**F(23)^3^ | 0.90 | 1.86 | 2.744(8) | 168 |
| N(6)-H(6C)**···**O(2)^4^ | 0.90 | 1.81 | 2.683(8) | 163 |
| N(7)-H(7C)**···**O(1)^2^ | 0.90 | 1.83 | 2.720(8) | 170 |
| N(7)-H(7D)**···**F(15)^2^ | 0.90 | 1.82 | 2.707(10) | 170 |
| C(1)-H(1A)**···**O(14)^2^ | 0.97 | 2.35 | 3.165(14) | 142 |
| C(5)-H(5)**···**O(2)^1^ | 0.93 | 2.57 | 3.419(8) | 152 |
| C(11)-H(11B)**···**O(1)^1^ | 0.97 | 2.56 | 3.342(9) | 137 |
| C(15)-H(15A)**···**O(5)^4^ | 0.97 | 2.48 | 3.395(11) | 157 |
| C(15)-H(15B)**···**O(7)^3^ | 0.97 | 2.54 | 3.444(11) | 155 |
| C(27)-H(27B)**···**O(9)^2^ | 0.97 | 2.60 | 3.324(14) | 132 |
| C(1)-H(1B)**···**F(8)^2^ | 0.97 | 2.54 | 3.250(10) | 130 |
| C(9)-H(9)**···**F(17)^5^ | 0.93 | 2.45 | 3.364(10) | 166 |
| C(10)-H(10A)**···**F(8)^1^ | 0.97 | 2.54 | 3.261(10) | 131 |
| C(10)-H(10B)**···**F(24)^4^ | 0.97 | 2.53 | 3.238(10) | 130 |
| C(11)-H(11A)**···**F(24)^4^ | 0.97 | 2.49 | 3.271(8) | 138 |
| C(12)-H(12A)**···**F(14)^1^ | 0.97 | 2.42 | 3.340(10) | 159 |
| C(12)-H(12A)**···**F(18)^1^ | 0.97 | 2.43 | 3.121(9) | 128 |
| C(13)-H(13B)**···**F(20)^1^ | 0.97 | 2.37 | 3.200(10) | 143 |
| C(13)-H(13B)**···**F(23)^1^ | 0.97 | 2.42 | 3.119(8) | 129 |
| C(21)-H(21)**···**F(16)^2^ | 0.93 | 2.49 | 3.305(9) | 147 |
| C(24)-H(24B)**···**F(13)^1^ | 0.97 | 2.38 | 3.276(10) | 153 |
| C(25)-H(25A)**···**F(8)^2^ | 0.97 | 2.35 | 3.220(10) | 149 |
| C(25)-H(25B)**···**F(18)^2^ | 0.97 | 2.45 | 3.229(10) | 137 |
| C(26)-H(26A)**···**F(11)^2^ | 0.97 | 2.41 | 3.146(11) | 132 |
| 1. x, y, z; 2. x, -1+y, z; 3. 1-x, -y, 1-z; 5. -1+x, -1+y, z. | | | | |

**Figure 1S:** HRMS spectra of complex **1**.

**Figure 2S:** ^1^H NMR spectra of complex **1** in DMSO-d_6_ at 25 ºC.

**Figure 3S:** HRMS spectra of complex **2**.

**Figure 4S:** ^1^H NMR Spectra of Complex **2** in DMSO-d_6_ at 25 ºC.

**Figure 5S:** HRMS spectra of complex **3**.

**Figure 6S:** ^1^H NMR spectra of complex **3** in DMSO-d_6_ at 25 ºC.
